# Rifabutin: A Repurposing Candidate for *Mycobacterium abscessus* Lung Disease

**DOI:** 10.3389/fmicb.2020.00371

**Published:** 2020-02-27

**Authors:** Thomas Dick

**Affiliations:** ^1^Center for Discovery and Innovation, Hackensack Meridian Health, Nutley, NJ, United States; ^2^Department of Medical Sciences, Hackensack Meridian School of Medicine at Seton Hall University, Nutley, NJ, United States

**Keywords:** rifabutin, *Mycobacterium*, repurposing, lung disease, *erm41*

*Mycobacterium abscessus* (Mab) is a fast-growing cousin of the infamous *Mycobacterium tuberculosis*. Both bacteria cause difficult-to-cure lung disease. In contrast to the slow-growing obligate pathogen *M. tuberculosis*, Mab is an opportunistic pathogen. Ubiquitously present in soil and water, Mab typically causes disease in vulnerable populations, including immune-compromised patients and people suffering from lung disorders such as cystic fibrosis and chronic obstructive pulmonary disease. Whereas, a drug regime that cures tuberculosis within 6 months is available, cure rates for Mab with the currently recommended combinations are 50% at best (Kwak et al., 2019). The prevalence of Mab pulmonary disease is increasing and more efficacious drugs are urgently needed (Wu et al., 2018; Daniel-Wayman et al., 2019).

A key drug in the regimen against tuberculosis is rifampicin. Inclusion of this rifamycin in anti-tuberculosis therapy in the 1960's resulted in dramatic treatment shortening and formed the basis of the 6-month curative regimen still in use today (Ganapathy et al., 2019). However, rifampicin is not used clinically for the treatment of Mab disease due to its poor *in vitro* potency. Instead, the cornerstone of Mab disease therapy is a macrolide, either azithromycin or clarithromycin. It is plausible that the lack of a rifamycin in Mab regimens contributes to unfavorable outcomes (Ganapathy et al., 2019).

In a screen of approved drugs against Mab, we were surprised to find that the rifampicin analog rifabutin (Crabol et al., 2016) was active *in vitro* (Aziz et al., 2017). The higher activity of rifabutin is likely due to differences in mycobacterial cell pharmacokinetics: rifampicin, containing a hydroquinone moiety, appears to be more readily metabolized by Mab than rifabutin. However, the mechanism underlying the differential potency remains to be determined (Ganapathy et al., 2019). Importantly, it was demonstrated that rifabutin is bactericidal against Mab and active against all three Mab subspecies (Aziz et al., 2017). Furthermore, no antagonistic effects with other clinically used anti-Mab antibiotics were found (reviewed in Ganapathy et al., 2019).

To provide additional preclinical data supporting repurposing of rifabutin, we recently measured its efficacy in a murine model of Mab lung disease. Rifabutin was as efficacious as the first line drug clarithromycin, both administered at the mouse equivalent of their clinically approved doses. As expected, rifampicin lacked efficacy (Dick et al., 2019).

Interestingly, *in vitro* results are emerging suggesting that rifabutin is not only active as a single agent, but also appears to suppress inducible macrolide resistance, an intrinsic resistance mechanism frequently encountered in Mab isolates (Nash et al., 2009). Co-treatment of macrolide resistant Mab with rifabutin and clarithromycin showed a synergistic effect. Rifabutin being an inhibitor of transcription appears to prevent effective transcriptional induction of the resistance-mediating *whiB7-erm41* system (Hurst-Hess et al., 2017; Aziz et al., 2020). Thus, rifabutin may hold macrolide resistant Mab in a macrolide susceptible state. If confirmed *in vivo*, this finding, would support a one-two punch attack against the infection.

Concomitant to these encouraging preclinical data, a first clinical success story was recently reported (Cheng et al., 2019). Immune-compromised patients (producing antibodies against their own IFN-γ) who suffered from refractory disseminated Mab disease were treated with rifabutin-based combinations and followed up clinically as well as by imaging. The authors conclude that “Rifabutin is an oral agent that can be effectively combined with azithromycin in long-term maintenance regimens against Mab in immunodeficient adults. Adverse effects are frequent early on; however, re-challenge appears to be safe and outcomes favorable” (Cheng et al., 2019).

Our preclinical results, together with these early clinical data suggest that rifabutin may improve outcomes of refractory Mab disease. Just as inclusion of a rifamycin was a game changer in the treatment of tuberculosis, rifabutin may both improve cure rates and reduce treatment duration of largely incurable Mab lung disease.

## Author Contributions

The author confirms being the sole contributor of this work and has approved it for publication.

### Conflict of Interest

The author declares that the research was conducted in the absence of any commercial or financial relationships that could be construed as a potential conflict of interest.

## References

[B1] Aziz D. B. Go M. L. Dick T. (2020). Rifabutin suppresses inducible clarithromycin resistance in *Mycobacterium abscessus* by blocking induction of whiB7 and erm41. Antibiotics 9:72. 10.3390/antibiotics9020072 32050554 PMC7168051

[B2] Aziz D. B. Low J. L. Wu M. L. Gengenbacher M. Teo J. W. P. Dartois V.. (2017). Rifabutin is active against *Mycobacterium abscessus* complex. Antimicrob. Agents Chemother. 61, e00155–e00117. 10.1128/AAC.00155-17 28396540 PMC5444174

[B3] Cheng A. Sun H. Y. Wu U. I. Sheng W. H. Chen Y. C. Chang S. C. (2019). Disseminated *Mycobacterium abscessus* infections in patients with neutralizing anti-interferon-gamma autoantibodies treated with Rifabutin-based combination regimens. Open Forum Infect. Dis. 6, S492–S493. 10.1093/ofid/ofz360.1224

[B4] Crabol Y. Catherinot E. Veziris N. Jullien V. Lortholary O. (2016). Rifabutin: where do we stand in 2016? J. Antimicrob. Chemother. 71, 1759–1771. 10.1093/jac/dkw024 27009031

[B5] Daniel-Wayman S. Abate G. Barber D. L. Bermudez L. E. Coler R. N. Cynamon M. H.. (2019). Advancing translational science for pulmonary nontuberculous mycobacterial infections. A road map for research. Am. J. Resp. Crit. Care Med. 199, 947–951. 10.1164/rccm.201807-1273PP 30428263 PMC6467310

[B6] Dick T. Shin S. J. Koh W. J. Dartois V. Gengenbacher M. (2019). Rifabutin is active against *Mycobacterium abscessus* in mice. Antimicrob. Agents Chemother. 64:e01943-19 10.1128/AAC.01943-19 31767722 PMC6985736

[B7] Ganapathy U. Dartois V. Dick T. (2019). Repositioning rifamycins for *Mycobacterium abscessus* lung disease. Expert Opin. Drug Disc. 14, 867–878. 10.1080/17460441.2019.1629414 31195849 PMC6663560

[B8] Hurst-Hess K. Rudra P. Ghosh P. (2017). *Mycobacterium abscessus* WhiB7 regulates a species-specific repertoire of genes to confer extreme antibiotic resistance. Antimicrob. Agents Chemother. 61, e01347–e01317. 10.1128/AAC.01347-17 28874378 PMC5655061

[B9] Kwak N. Dalcolmo M. P. Daley C. L. Eather G. Gayoso R. Hasegawa N. (2019). *Mycobacterium abscessus* pulmonary disease: individual patient data meta-analysis. Eur. Respir. J. 54:1801991 10.1183/13993003.01991-2018 30880280

[B10] Nash K. A. Brown-Elliott B. A. Wallace R. J. Jr. (2009). A novel gene, *erm*(41), confers inducible macrolide resistance to clinical isolates of *Mycobacterium abscessus* but is absent from *Mycobacterium chelonae*. Antimicrob. Agents Chemother. 53, 1367–1376. 10.1128/AAC.01275-08 19171799 PMC2663066

[B11] Wu M.-L. Aziz D. B. Dartois V. Dick T. (2018). NTM drug discovery: status, gaps and the way forward. Drug Disc. Today 23, 1502–1519. 10.1016/j.drudis.2018.04.001 29635026 PMC6078814

